# Epigenetic reprogramming in the epithelial-to-mesenchymal transition

**DOI:** 10.1186/1753-6561-6-S6-O28

**Published:** 2012-10-01

**Authors:** Stephen Hoang, Marcin Cieślik, Sanjay Chodaparambil, Natalya Baranova, Manish Kumar, David Allison, Jake Wamsley, Lisa Gray, Marty Mayo, Stefan Bekiranov

**Affiliations:** 1 Department of Biochemistry and Molecular Genetics, University of Virginia School of Medicine, VA, USA

## 

The epithelial-to-mesenchymal transition (EMT) is a cellular dedifferentiation process that is critical to development, wound healing and metastasis. Like other cell state transitions, such as differentiation, EMT is accompanied by genome-wide epigenetic reprogramming. However, the relationship between reprogramming and functional changes in the cell is poorly understood. In an A549 non-small cell lung cancer EMT model system we observed changes in chromatin state between epithelial and mesenchymal states. Multivariate analyses were applied to paired (epithelial and mesenchymal) ChIP-seq data for 18 histone modifications/variants and expression microarray data. We observed epigenetic co-regulation of genes associated with EMT, as well as their proximal enhancers. We also observed epigenetic activation or repression of functionally distinct sets of enhancers. These genes and enhancers are regulated and bound by a small set of transcription factors, specifically AP-1, NF-κB and c-Myc. These transcription factors themselves also a show an epigenetic profile similar to the EMT-related genes. Together, these observations suggest a chromatin-mediated transcriptional feedback mechanism that establishes and maintains the phenotypic switch.

